# A Memoir on the Yellow Fever of the West Indies, *as It Occurred in the Year* 1838, *at St. Pierre, Island of Martinique*

**Published:** 1840-02-15

**Authors:** E. Rufz

**Affiliations:** Adjunct Professor at the School of Medicine of Paris


// MEDICAL EXAMINER.

DEVOTED TO MEDICINE, SURGERY, AND THE COLLATERAL SCIENCES.

No. 7.] PHILADELPHIA, SATURDAY, FEBRUARY 15, 1840. [Vol. III.

### A MEMOIR ON THE YELLOW FEVER OF THE WEST INDIES,

*As it occurred in the year* 1838, *at St. Pierre, island of Martinique.* By E. Rufz, D.M.P., Adjunct Professor at the School of Medicine of Paris.

Translated for the Medical Examiner, from the French Manuscript.—[*Continued.*]

### VARIETIES OF THE DISEASE.

Under this title I have arranged some cases, which, either in the duration and progress of the disease, or in certain symptoms, or in some peculiarity in their mode of termination, differed from the majority,—but which, nevertheless, cannot be considered otherwise than as varieties of the epidemic. Such is the following one.

1st. Mademoiselle Eliza, a milliner of Coblentz, twenty-six years of age, of a strong constitution, and very sanguine temperament, came to the colony three months before. She always led a very sober life; but, suffering from heat, she usually sought a current of air, and even placed herself at her window during the night. In the beginning of February she contracted a pulmonary catarrh, cough, difficulty of breathing, uneasiness and weight about the head, and loss of appetite.

On the 5th of February I bled her. The symptoms, however, instead of diminishing, increased; the cough became more frequent; the head was constantly oppressed; the appetite disappeared; the tongue became whitish, with a yellowish coating over nearly the whole surface, and red at the tip.

On the 19th she took a lemonade, with two ounces of cassia. This did not produce any evacuation. The cephalalgia became intolerable. Her face was very red, and there was considerable uneasiness, with a contusive pain in the limbs; slight delirium; pulse one hundred, and full; skin warm.

Sixteen ounces of blood were taken from her in the evening, after which she felt better; the cephalalgia was much less, and she was more calm at night; the cough, which had persisted until this time, diminished; she had three stools; pulse one hundred and four, and regular; skin hot, and slightly moist.

Emollient drinks, injections, pediluvia, and embrocations of lemon juice, were made use of.

Slight epistaxis occurred in the course of the day.

On the 21st the symptoms were nearly the same, and of the same intensity. There was no suppression of urine; stools were procured by enemata. There was a recurrence of the epistaxis; and she complained of some twitchings, and soreness of the tendons, which I attributed to the embrocations. I, therefore, discontinued them.

22*d.*—Epistaxis; patient in the same condition; pulse still one hundred and four.

23*d.*—Had passed a restless night; there was stoppage of the nose, without epistaxis, and deafness; her eyes were very bright; she had a fulness of the head, but no cephalalgia; spoke abruptly; had slight oppression, but no cough; her anxiety was greater; her skin warm; her pulse one hundred and four, harder, and more corded. She had no evacuation from the bowels, but passed her water readily.

Thirty leeches were applied behind her ears; her deafness diminished for some hours, but afterwards returned.

From this day her symptoms were aggravated; her deafness was more marked, but never total. She was slightly delirious, especially during the night, but had no cephalalgia; her decubitus was dorsal. She was indifferent to what was passing around her, but nevertheless pretty calm. Her cheeks flushed and grew pale, alternately, especially during the days of the 27th and 28th.

Her tongue, which, from the commencement, had had more than three-fourths of its surface coated, with a small part at the tip red, and had been all the time moist, cleaned a little on the 28th.

She had neither thirst nor nausea, except on the 28th, after taking two spoonfuls of castor oil.

Abdomen free from pain, but a little meteorized from the 27th to the 30th.

The 26th, 27th, and 28th, there was suppression of urine. During from twenty-four to twenty-eight hours, between the 28th February and the 1st March, her urine was scanty, and flowed involuntarily.

Up to the 27th she had had no stools, except after the use of enemata. On this day, while her face flushed and grew pale alternately, she had suddenly two or three evacuations of sanious, black, fœtid blood, which might be estimated at about a pound.

The 28th her stools were still bloody, but less in quantity, and thicker. I administered two spoonfuls of castor oil, with three of almond oil; the character of the stools was changed, and they became yellowish.

Her pulse was accelerated, beating from one hundred and twenty-four to one hundred and thirty-two; it was regular, but feeble; skin moderately warm, without perspiration.

On the 23d, thirty leeches had been applied behind the ears; on the 27th, thirty to the epigastrium, and the same number behind the ears; on the 28th, thirty to the hypogastrium; on the night of the 27th, a blister to each thigh; diluent drinks and enemata were directed.

From the 28th February to the 6th March, there was no change in her condition. Her nights were calm, without sleep; there was an alternation of paleness and flushing of the face; very marked deafness, and stupor; her understanding was nevertheless clear. Decubitus dorsal; tongue slightly coated at its base, and red at its tip; abdomen less meteorized; stools of a yellowish colour, procured by injections; urine scanty, clear, and under the control of the will; her pulse had fallen from one hundred and thirty-two to one hundred and twenty-four, and was regular; skin moderately warm, and covered from time to time with a clammy sweat.

From the 6th of March her amelioration was evident, but very slow. There was no icterus; but her deafness, as well as her stupor, persisted for more than fifteen days. The patient had, during the first few days after she commenced to rise from her bed, a tremour in all her limbs,—and her intellect, although clear, appeared to be weakened, for she often laughed without a cause. Her appetite and digestion were perfectly re-established, but many precautions were necessary. Those who were about the patient, finding that things did not go on fast enough, took it in their heads, on the 10th of March, to give her an oleaginous mixture, which she threw up, without being otherwise incommoded by it.

During the remainder of her convalescence, there was a miliary eruption over her whole body. She recovered very slowly.

This case closely resembles that of Mdlle. Alexandrine Giron, No. 3; it differs in many respects from the others which I have made use of, to establish the main parts of this memoir.

At first the disease was slow in its progress. The period of calm, so remarkable in yellow fever, was wanting. In fine, the course of the disease was entirely different.

The other cases of yellow fever terminated in the course of the first week: this continued grave during from seventeen to twenty days. It presented many symptoms which do not ordinarily exist, viz., deafness, carphologia, &c.

It is probably this form of the disease which has been considered as *gastro-entero-cephalitis*, by M. Vatable, physician to the king at Guadaloupe, in 1826. (Annales Maritimes, 13th year, 1828, No. 3.)

I did not have an opportunity of examining the anatomical lesions, to see if they were also different. But as this affection attacked Europeans who had recently come to the colony, and in the midst of the epidemic, I have thought that it ought to be referred to yellow fever, of which it should be considered a variety. I have seen no similar affection at other times.

This case, and that of Mdlle. Giron, are the only two of the kind that I have seen. It is worthy of notice, that in both cases the persons affected were females.

2d. I have also considered as a variety of yellow fever, the severe fevers which attack the natives of the country, while the yellow fever is prevailing. All who have written on the yellow fever of the colonies have confirmed this fact; but, during the last epidemic, it was more strikingly the case, the number of creoles attacked being much greater. Here is a remarkable example of it, of which one of our brother physicians was the subject.

CASE.

Charles Seisson, a physician, thirty-five years of age, of rather a feeble than strong constitution, a creole of Martinique, who had passed eleven years in France, but returned to the island six years ago. He led a very temperate life; he, however, frequently complained of palpitation of the heart. On the 11th of February, he was wet by a rain while going into the country. In the evening he had a chill, followed by heat and vomiting; during the night restlessness, fever, and headach. He was seen next day by Dr. Noverre.

*12th February.*—Considerable headach; eyes and face injected; skin warm; pulse frequent, full, and regular; no nausea; abdomen tense; no evacuation of the bowels; general uneasiness. Forty leeches were applied to the neck, and twelve ounces of blood taken from the arm. The cephalalgia diminished after the bleeding, but the fever continued of the same intensity.

*13th.*—Had passed a bad night; was in nearly the same state. Thirty leeches were applied to the anus.

*14th.*—Restlessness, and great anxiety on the part of the patient, about the termination of his disease; skin warm; no remission in the progress of the symptoms. Four spoonfuls of oil were administered; this purgative produced some nausea, and was followed by six stools.

*15th.*—Continuance of the fever, and restlessness. The patient felt no definite pain. At noon thirty leeches were applied to the epigastrium. About 2 o'clock he began to grow cold, and lipothymia ensued. (The leech-bites did not bleed much.) At 5 o'clock I was called in consultation.

The patient's face was of a dark red, his conjunctivæ injected, and the expression of his countenance savage; he did not recognise me. His forehead was warm, but his limbs were cold, and his pulse imperceptible. The muscles of the abdomen were tense, and pressure on them caused the patient to start. No vomiting; no evacuation, either of the bowels or bladder; no sweat. Some twitchings of the tendons were soon added to these symptoms. Since 2 o'clock, sulphate of quinia, and injections of cinchona, were administered, with blisters, sinapisms, and embrocations. At 5 o'clock he was seized with convulsions, and at 6 he died.

An autopsy was made on the 16th, at 10 in the morning. Condition of the exterior of the body:—Greenish yellow, with purple ecchymoses on the back of the neck, and in all dependent portions; rigidity such as follows death.

The skin was sticky, and, together with the dura mater, very much injected. The longitudinal sinus was filled with black blood, fluid, and without coagulum.

*The brain.*—The pia mater, at its base, as well as on its convex surface, was very much injected; it was easily detached from the substance of the brain. At its posterior part, there was a slight effusion of serum under the arachnoid membrane. The glands of Pacchioni were very much developed. The substance of the brain was somewhat soft, without, however, its texture being changed in any respect. Both portions were finely injected with black blood. The ventricles contained two spoonfuls of reddish serum.

The lungs were well formed, and presented no adhesions with the pleuræ; their tissue was well filled with air. The inferior lobes of both sides were of a darker colour, in consequence of the stasis of the blood in these dependent portions. There was no where either effusion of blood, or hepatization in the pulmonary tissue itself. These lungs might be considered as being perfectly healthy.

The heart was larger than it usually is in a man of the age and size of Dr. Seisson. This augmentation might be estimated at one-fourth; it was principally owing to the dilatation of the two ventricles. The parietes of the left ventricle were three and a half lines thick. The orifices of the cavities of the heart offered no sign of ossification, or other organic alteration.

The blood contained in the ventricles, auricles, and in the aorta and vessels generally, was black and very fluid, and contained no coagulum.

The pleuræ and pericardium contained a scarcely appreciable quantity of serum.

The cavity of the peritonœum was free from effusion, or any other alteration. The stomach was very much distended and contained about a tumblerful of very thin black blood. Its mucous membrane was stained by the blood contained in it; but even after it was washed, it preserved a general red tint, without any distinct arborescence; it had no coating of mucus, and was fragile; but it was not softened, by which I mean, that if it was attempted to detach shreds of it, these shreds were shorter than they ordinarily are, and broke more easily.

The duodenum and the whole of the small intestines were coated with an abundant grayish yellow, and rather adhesive mucus. Beneath this layer of mucus, the mucous membrane, particularly in the duodenum, and towards the termination of the small intestines, presented injections of various forms and dimensions, and generally of a bright red. This mucous membrane, like that of the stomach, was more fragile than it is in its natural state.

In the jejunum, the glands of Brunner were prominent, whitish, and as large as millet seed.

The glands of Peyer were neither enlarged, nor ulcerated; they were difficult to distinguish.

The large intestines offered, especially in the rectum, a bright injection, similar to that of the small intestines. The mucous membrane of this portion was fragile, but not actually softened. The glands were not visible, and there was no other alteration.

The mesenteric glands were larger than they ordinarily are, by a fourth. They were hardly tinged with red, and were not softened.

The liver, considered in a general way, was not enlarged; it did not extend beyond the false ribs. It contained little blood, and its tissue was very hard and firm, being almost in the commencement of cirrhosis. It was of a brick-red colour. The two portions, yellow and red, were distinct; the yellow seemed to predominate. The capsule of Glisson was yellow. The gall bladder, viewed exteriorly, was almost semitransparent, and considerably distended; it was filled with clear bile, which was serous, whitish, and very fluid. The mucous membrane lining it was the seat of a remarkable red injection, in the form of rounded spots, which bore some resemblance to the marks left by a very recent attack of variola. This injection was no where arborescent. There was no mucous in the mucous membrane, which could be raised in long shreds of considerable strength.

Some of the glands situated along the course of the biliary ducts, were double their ordinary size, hard and yellowish, and crushed under the fingers; they were in a truly morbid condition.

The spleen was a third larger than we usually find it, and was filled with black blood; its tissue was firm.

The kidneys were of a deep colour, and were filled with blood, but were not modified in their structure. The bladder bent upon itself, contained some turbid urine, full of mucus. Its mucous membrane was pale and smooth.

All the tissues, when cut, yielded very fluid blood of a black colour.

We will not lay any stress on the lesions which evidently occurred previously to the disease, (those of the heart and liver.) The case of Seisson resembled very much that of Mad. Clozet. It would be called a case of congestive fever. But although this course and this termination are not usual in yellow fever, is it possible not to perceive the greatest analogy between affections which are equally attended by black matter in the stomach, icterus after death, and alteration of the biliary ducts, of the blood, and of the whole intestinal canal?

In my own practice, I can enumerate thirty-eight persons, either born in the country, or acclimated for ten or fifteen years, who, nevertheless, were attacked by the disease. All the physicians agree in making the same observation. These persons, however, had the disease more mildly. Among thirty-eight patients of this description, but two deaths occurred.

*3dly.*—In the months of May and June, the disease, in a majority of cases, presented differences enough to constitute another variety still. Here is an example of it.

*Case.*—M. Second, of the ship Neptune, twenty-three years of age. He was very well on the 19th of May. In the evening, while returning to the ship, he fell overboard and got wet. In the night he was taken with a severe chill, which was quickly followed by a high fever, with a contusive pain in the limbs, and also a pain in the loins.

On the morning of the 20th, I found him in the following state: cephalalgia intense; pupils dilated; face highly coloured, intellect somewhat dull; contusive pain in the limbs. He complained of a pain in the epigastrium; his pulse was at fifty-four, and his skin preserved its natural state without any morbid degree of warmth. Towards evening there was superadded to these symptoms, a restlessness very remarkable on account of its being at variance with the state of the pulse, and temperature of the skin. He was bled twelve ounces, the blood being of a very dark colour, and two blisters were applied to his legs.

He passed the night well enough; the cephalalgia was somewhat diminished; pulse sixty-four: restlessness and pain in the epigastrium. Thirty leeches to the epigastrium and a purgative enema. From this moment the patient got better and better, and on the 26th he was able to return to his ship.

He enjoyed good health until the 13th of June, when he had a relapse nearly similar to the first attack, which lasted eight days. He recovered, but some days after he showed symptoms of mental derangement. All these symptoms disappeared, and he left for France perfectly cured. He was bled five times.

At the time of making these observations, I had three other cases of a similar nature, that is to say, that the symptoms of the first period, which, at the commencement of the epidemic, lasted at least a day, only showed themselves for one, or at the most two, hours, and were replaced by those of the second period, (called *period of calm.*)

This form was less grave, and more under the control of medicine.

I learn from M. Catel that he had made the same observation at the hospital, after seeing a much greater number of patients.

It is also at this period of the epidemic, that hæmorrhages from the nose and mouth recommenced to appear, as I have already said, and appeared as favourable signs.

Lastly, I shall describe as another variety of the epidemic, that which attacked the children. Almost all in the city were affected. In my own practice, from October to July, I counted as many as eighty-four. Of this number, I lost but two; the disease then was less severe than among the Europeans. The two who succumbed were brothers. Here is the account of one of them.

*Case.*—A child named Page,* eight years of age, of a moderately stout frame, but very lymphatic, having a fair semitransparent skin, and slender muscles. His health, however, was habitually good. On the 12th of April he was sprightly, and ate with a good appetite. During the night of the 12th he was restless, and when he awoke the next morning, he complained of great pain in the head; skin hot, pulse frequent, face flushed; he vomited twice. They gave him immediately some cassia, both by the mouth and rectum, which produced three evacuations.

I saw him on that day, at eleven o'clock in the morning. Cephalalgia with coma; eyes injected; skin warm; pulse one hundred and twenty, and regular; abdomen yielding, and free from pain; no pain in the epigastrium; a free flow of urine. The night of the 13th there was restlesness; four stools; urine rather scanty; the stupor appeared to be a little diminished; but as the day advanced, this symptom increased; three stools during the day; pulse one hundred and twenty; some twitchings of the limbs, and of the muscles f the face; urine very scanty.

At three o'clock, twenty leeches were applied to the epigastrium, and two blisters to the legs. His face became very pale after the loss of blood; the coma more profound, and the pulse more frequent, rising to a hundred and twenty-eight.

From this time to that of his death, which took place on the evening of the 16th, the coma continued without intermission, combined, however, with great restlessness; the child lay in every position on his bed, without keeping any one for a long time.

From time to time he had some twitching of the tendons and even a little carphologia. He frequently opened his eyes; his look was fixed, and ended with a slight strabismus.

When questions were put to him in a loud voice, he answered as if he had been awaked from a sleep; his answers were not always straight, and he was even delirious.

* This child's mother was a teacher. She had lost, fifteen days before, his brother, six years of age, who died of the same affection; these children, however, were the only two in the establishment that were sick.

On the 16th, he had a slight pain during deglutition. He retained his strength to the last moment in a remarkable degree; he could raise himself to a sitting posture, and keep it quite firmly.

His abdomen, during the whole time, was yielding and free from pain; his urine continued very scanty: he passed at the most, one or two spoonfuls in twenty-four hours. His stools, after enemata of cinchona and assafœtida, increased, (there were eight or nine on the 16th) and contained some clots of blood. From the 15th, the heat of skin was wanting, and the extremities grew cold; this coldness went on increasing continually; the pulse also became imperceptible thirty-six hours before death. The skin assumed a slightly jaundiced tint.

The child died on the 16th, at ten o'clock in the evening. During the first two days, cinchona, assafœtida, and camphor were tried.

The autopsy was made at eight o'clock on the morning of the 17th. The body was of a very pale yellow, with some purple colouration on dependent parts; the longitudinal sinus contained fluid blood without a coagulum. The meninges, though examined with the greatest care, showed no morbid injection; they were rather pale, and were easily detached from the brain. There was no serous infiltration under the pia mater. The ventricles of the brain contained only two tea-spoonfuls of serum. Both the substances of the brain were firm, and moderately injected. The lungs were perfectly healthy; the bronchi pale. The pleuræ and pericardium contained no effused serum. The heart was soft and flaccid, and contained some fluid black blood, without coagulum. The aorta was not coloured.

The stomach was little dilated, and contained about two ounces of a black matter, resembling coffee grounds, but no mucous; its colour was generally whitish, interspersed with very fine red points, which looked like little dots under the mucous membrane. There was neither *ramollissement*, nor thickening.

The consistence of the mucous membrane was, however, somewhat fragile.

The small intestines were coated with an abundant grayish matter, and offered here and there patches of black matter like that which was found in the stomach. Their mucous membrane was generally whitish; but here and there was seen a minutely punctated colouring, similar to that of the stomach. It was of a fragile consistence, especially near the cœcum.

The glands of Brunner were quite prominent throughout the whole extent of the intestine; but the glands of Peyer were not visible.

The large intestines presented a grayish matter, and a more deeply injected appearance than the small intestines. This injection was not arborescent, but disposed in uniform patches like an ecchymosis. Its consistence was good, but somewhat fragile; the shreds of the membrane were only half an inch long.

The mesenteric glands were firm and of a whitish colour, and a little more developed than they commonly are.

The liver was of its normal size; but it was yellow like Spanish tobacco; this colouring was uniform; the two substances of which it is composed, could not be distinguished. Its tissue was of a firm consistence, and a little sticky.

The gall-bladder contained a small quantity of blackish and rather fluid bile; its mucous membrane was healthy and showed no injection.

The spleen was firm and of its ordinary size.

The kidneys were unchanged. The bladder offered an injection which was decidedly arborescent, and had its seat in the submucous tissue. The mucous membrane afforded very long shreds. This injection appeared to be inflammatory. (The child had had three blisters applied.)

Notwithstanding the differences which the history of these symptoms present to us, I think that one would be struck with the perfect similarity between the anatomical lesions found in this child, and those which we have before seen to exist in adults.

The other cases which I had to treat, were not so grave. In almost all of them, the disease made its appearance very suddenly; at its commencement, the symptoms were very marked at all hours of the day;—intolerable headach, chill, heat, high fever, often vomiting, more or less frequently repeated, convulsive movements, and, in four cases of children, actual convulsions during a quarter or half hour. During the first few hours, in connection with the fever, succeeded a drowsiness, from which nothing could rouse the patient; the children, however, retained their intellectual faculties. The eyes were injected, the pupils dilated, face flushed; a little thirst, anorexia; abdomen yielding, and free from pain; no stools.

These symptoms, except in four cases, disappeared after a lapse of thirty-six or forty-eight hours, and the children immediately entered a state of convalescence. Their faces, however, were pale, shrunken, and more altered than any one would imagine they could be, after so short an illness.

About a third vomited worms; in these cases the vomitings were sometimes preceded by *malaise*, pallor, agitation, pain in the epigastrium, and a countenance expressive of distress, but the worms were frequently voided by stool. One child passed thirteen. In these cases, although our attention was directed to the subject, we were unable to note any particular symptom as revealing the presence of these animalcules in the primæ viæ.

Of three very severe cases, which were all that occurred in my practice, two proved fatal, one was cured. The most conspicuous symptoms were continued drowsiness, and fever without remission, and in the two fatal cases, black vomiting during the last two days, precisely similar to the black vomit of yellow fever. I learn from my brother physicians that, in the fatal cases which came under their notice, the vomiting of black matter also existed.

As for the anatomical changes, there has already been seen, in the case of little Page, an injection of all the organs, and a yellowish discolouration of the liver. In another autopsy, which I had occasion to make, in company with M. Boulin, we found the membranes on the convex portion of the brain, almost colourless; but the large vessels at its base were very highly injected, and there was an effusion of blood to the amount of nearly three spoonfuls in the lower portion of the arachnoid cavity. This blood was black, contained a little serum, and coagulated on exposure to the air. The substance of the brain was firm. The lungs and the heart contained some black blood.

The stomach and intestines were pale, and their mucous membrane somewhat fragile; but they did not exhibit any particular lesion.

The spleen was in its natural state; but the liver was yellowish, and there was no distinction between the two portions which enter into its composition: it retained its ordinary volume. The gall-bladder was moderately dilated, and contained a spoonful of bile, of a dark greenish colour, and very fluid; its mucous membrane was not injected.

The body exteriorly, presented a yellow appearance, which was rather the result of discolouration, than of icterus.

This child was an European, seven years of age, had been in the colony eighteen months. He had been sick five days, with headach, coma, black vomit from the third day, delirium and convulsive movements; such had been the principal symptoms. They had put on him one hundred and fifty leeches, and had made applications of ice to his head.

I am of opinion that the city lost about fifteen children in this manner. This estimate is made from some data furnished by the other physicians of the city, and an examination of the reports of the civil authorities.

Generally the patients thus affected, were little boys; only twenty-two out of eighty were girls.

They were, with few exceptions, from three to twelve years of age, and the chief determining cause was a great degree of exposure to the sun, which agrees very well with the age and sex of the children.

It is impossible not to see a strong resemblance between this affection of children, and the yellow fever of Europeans. Might not this arise from a similarity in the state of their blood. Generally, the creole children, in early age, have a fresh complexion, almost as rosy as that of Europeans, but towards the age of eight years they grow thin and pale, and assume what is very well known here under the name of *creole complexion.* The same happens to Europeans after an attack of yellow fever, or a long residence in the colonies—they acquire the creole complexion. It is the seal of acclimation.

In the accounts of the preceding epidemics, I see no notice of children born in the country suffering with an affection closely resembling yellow fever, simultaneously with the occurrence of the latter disease among Europeans.

Those affected were principally white children; some mulattoes were among the number, but I saw no negro children who were attacked by the disease. This yet remains to be observed.

With some children, as with some adult creoles, the disease presented an intermittent type; that is to say, that, after a severe paroxysm which lasted from twenty-four to forty-eight hours, there was a notable apyrexia, followed by some paroxysms of a less severe character. These cases were not the most grave: the fever was arrested by sulphate of quinine.

There were seven or eight relapses which were not attended with any grave symptoms.

If we now consider the epidemic as a whole, we shall perceive very distinct phases.

From September to December, it prevailed principally in the garrison: almost all the soldiers there were attacked by it. But few cases could be cited in the city.

In December and January it was the city that furnished the greatest number of sick; the number in the hospital diminished.

Towards the end of January, there was a remission of the severity of the disease.

It seemed to regain its vigour in February.

From March to July it prevailed chiefly among the merchant ships: it is a remarkable thing, that until that time but very few of their men had been affected, although their number was quite considerable.

In March, the disease extended to Fort Royal; and from February there prevailed in the other parts of the island fevers of a grave character. Some remarkable deaths were related; among others, that of M. Desvouves, a young physician, a creole, who had resided ten years in France, and since his return to the island had practised two years at Lamentin. According to the accounts which I have been able to obtain, these fevers resembled very much that of St. Pierre.

At St. Pierre, the number of sick seemed to be dependent on certain days; that is to say, that on some days five or six fell sick at once, and after that there were no new cases. These inauspicious days were not marked by any particular circumstance. Many physicians attributed it to the westerly wind which prevailed at that time.

Having once shown itself in certain localities, the disease appeared to attach itself there, and many fell victims to it. There were houses for which it seemed to have a predilection. I will cite as an example the nunnery of St. Joseph. After having been exempt until March, this convent was then visited by the disease. In the space of one month, six nuns and many pupils were taken sick; two of the former died. There was nothing peculiar in these localities.

It was the same case with the shipping; many vessels were in the roadstead for fifteen or twenty days without having an individual on the sick list, but when one fell sick, many others followed. Many writers have pretended that it was at the opening of the hold, that the disease showed itself among the sailors. The ship Edward had come in ballast, having nothing in her hold but fine sand. The ship, *Les deux Amélies*, of Bordeaux, had discharged her cargo, and her holds had been open for more than twenty days, when the first man was taken sick aboard.

The concomitant affection with which children suffered, prevailed in the city throughout the whole duration of the yellow fever, but it was in April and March that its victims were most numerous.

### CAUSES.

We have already said that the yellow fever did not reach Martinique before it had visited Tampico, New Orleans, and Havana, in 1837, and Gaudaloupe in July, 1838. We have asked ourselves whether the yellow fever did not pursue a rout from west to east, as the cholera did from east to west.

*Importations.*—When the disease made its appearance at St. Pierre, in September, the stormy season had fully set in. Ships coming from Europe remained at Fort Royal. They did not return to St. Pierre until the 25th October. We had taken the precaution of quarantining, at Fort Royal, all the coasting vessels from the infected colonies. And yet the disease did not extend to Fort Royal until March, (four months after its appearance in the island,) but at St. Pierre it manifested itself in September.

Can any fact demonstrate more conclusively than this that the yellow fever is not imported in vessels.

The disease made its first appearance in the garrison, then spread to the city, and lastly, prevailed in the roadstead. It is then neither from any infected ships, nor any salt marsh, that it proceeded. The roadstead was attacked at the period of harvest, when there was a considerable arrival of ships. Therefore, in all epidemics, the most thickly peopled places are most liable to be affected by the disease. The cholera, which was confined to Marseilles for six weeks, appeared in Beaucaire at the time of the fair in that town.

The barracks, the hospital, and the houses of those first taken sick, were not the places nearest to the sea. These houses were widely separated from each other, and were frequently in the most elevated situations in the city, (see especially the case of M. Fraquet.)

These facts are in opposition to all ideas of its importation.

*Infection.*—Let us now examine the facts relative to its being infectious. Let us see if, when the disease had appeared at any point of the city, it was on this point that it first concentrated its force, in order to spread towards others.

The disease commenced in the garrison; but the first who were taken sick in the city, M. Fraquet at the fort, and M. Belligny at the anchorage, were at a distance from the barracks, and had had no communication with them.

Almost all the garrison were on the sick list, except thirty or forty men, out of about five hundred.

When all the soldiers had passed through it, there was a short truce, but in March, two hundred men having come from France, the garrison recommenced furnishing its contingent.

But at the hospital, even the wounded, the venereal patients, and those labouring under other complaints, were taken with the yellow fever in their beds, after those infected with this disease were brought to the house, which is a very remarkable thing. Almost all the persons employed in this establishment were attacked, although they had been acclimated for a long time. Of eleven *sisters of charity*, ten were taken sick, and seven died. M. Catel himself, though he had passed twenty years in the colonies, as many in Senegal as in Martinique, experienced the epidemic influence. So that the hospital, during six weeks, appeared to me to be truly the seat of infection, but the neighbouring houses were exempt. I have already said that several houses, among others the nunnery of St. Joseph, had been distinguished as being particularly afflicted; they were at a distance from the hospital.

As regards the ships, the disease having once shown itself among them, many became its victims in rapid succession. It did not, however, follow the order of their position, that is to say, that the second ship affected was not moored alongside of the first. I have said that it was not at the time of opening the hold that the disease occurred. Its occurrence could not be referred to any particular circumstance, for the ships which contained freight, and those which had come in ballast, were attacked without any distinction.

I am, then, inclined to think that during the prevalence of the epidemic, certain places might be considered to have been as it were the foci of infection, that the infection was confined to these places and did not extend around.

*Contagion.*—As regards its being communicated from one individual to another, I have seen nothing which can warrant a belief in this method of transmission. I have seen women nurse their husbands, and persons nurse their friends without contracting the disease. The following is a remarkable fact. The governor going from Fort Royal to St. Pierre, took with him the musicians of the regiment, (in October, 1838.) The latter, seven in number, were lodged in the barracks of the garrison. Eight or ten days after their return to Fort Royal, the seven musicians were taken with the yellow fever, and five of them died. But the disease stopped there, at that time, and it was not until March, three months after, that it invaded the garrison and city of Fort Royal. Nevertheless the musicians had been permitted to remain among the other soldiers in the barracks, as well as in the hospital; no precautions had been taken for the purpose of isolating them. Can any fact demonstrate more clearly than this, that the yellow fever is infectious, and not contagious.

*Meteorology.*—Let us now study the meteorological conditions that existed during the prevalence of the epidemic.

The thermometer stood as follows :

| | | Highest temperature. | Lowest temperature. |
|---|---|---|---|
| October | - | 33 degrees C. | 28 degrees C. |
| November | - | 33 " | 27 " |
| December | - | 32 " | 26 " |
| January | - | 31 " | 24 " |
| February | - | 33 " | 26 " |
| March | - | 34 " | 27 " |
| May | - | 34 " | 27 " |
| June | - | 33 " | 28 " |

The barometer varied from 760 to 766. The hygrometer from 70 to 100 constantly. This was the case during the whole time.

The pluviometer gave the following results:

| | | | |
|---|---|---|---|
| October, | 9 inches | 3 lines | of rain. |
| November, | 14 " | 2 " | " |
| December, | 5 " | 2 " | " |
| January, | 8 " | 6 " | " |

From February to June, it was dry for seventy days, during which time the pluviometer showed only one inch and six lines; but during the remainder of the month of June, there fell 9 inches 6 lines.

At the commencement of the epidemic, there occurred in October nine storms; but since then we have had but two, which took place in June.

On the 11th of January took place the earthquake, which has been described in all the newspapers. This dreadful event had not been announced by any precursory disorders of nature: it could have had no influence on the epidemic.

As for the indications of the barometer, we must pass them over, they vary so little in the torrid zone, that no importance can be attached to them.

If we now turn our attention to the thermometer, we will see that during this year it has indicated the usual temperature of the colonies during years in which they were exempt from the yellow fever. The inhabitants, however, complained that the cool season, from December to February, was not so pleasant this year, which they attributed to the prevalence of westerly winds.

The rains during the last winter did not exceed their ordinary quantity; they were conspicuous enough in November, but from February, to June there was an uninterrupted drought.

Therefore, whether the thermometer stood high or low, whether it rained or shone, whether it was warm or cold, the yellow fever prevailed with the same intensity, without its progress appearing to be in the least influenced by the seasons of the year.

Maker, in his report, thought that he had detected some influence on the part of storms. But although in October (a period in which this phenomenon is frequent in the colonies) we can enumerate nine, yet during the eight months following this, they scarcely occurred at all, and, notwithstanding, the yellow fever continued to prevail.

We cannot say the same with regard to the direction of the wind. Every one remarked that it blew from the west more constantly than it had done during previous years. A comparison of the meteorological tables confirms the common observation. Dr. de Verteuil of Trinidad, and M. Corneut of Guadaloupe, insist upon this fact. These westerly winds come from the upper part of the Gulf of Mexico, from the direction in which the yellow fever prevailed on the continent during the preceding years. However, I only give this as a remarkable fact, and not as an evident cause of the yellow fever.

*Sex.*—It is generally agreed, that the yellow fever attacks men more frequently than women.

It would, however, be difficnlt to establish this opinion through what has taken place in the colonies. The great number of men that come to this country, sailors, soldiers, and small traders, bears no comparison with the small number of women. We can hardly enumerate any of the latter. If I were guided by an approximation, based upon my acquaintance in the city, I should say that the yellow fever attacked as many women (comparatively speaking) as men. I attended seven women; three were slightly affected, four gravely; one of the latter died. I have already said, that at the hospital, out of twelve sisters of charity, eleven were taken sick, and six died. At the convent of St. Joseph, there were eleven sick, of whom two died. These facts would make out that the disease was more severe with women than with men.

*Age.*—I must make the same observation with regard to age, that I made with regard to sex: few children or old men come to the colonies from Europe. To supply the deficiency of my own experience, I interrogated the natives who, on account of their intelligence or character, seemed to be worthy of confidence, and they all assured me that they had rarely seen old men attacked by yellow fever, while many instances were mentioned of children who suffered the preceding epidemics. I have myself reported one case of this kind.

I have heard of a man who died of yellow fever at the age of sixty, during this epidemic. He was a surgeon dentist, named Toby, who was travelling for pleasure. He insisted upon treating himself homœopathically. I could not ascertain how many billionths of a grain he had taken. The greater portion of those attacked were from 25 to 40 years of age.

*Acclimation.*—The period of acclimation is the condition which, above all others, deserves to be taken into consideration. The yellow fever not having existed at Martinique since 1826, we saw, at the commencement of this epidemic, many persons attacked by the disease, who had been in the island for three, seven, or ten years; but these were not so severely sick as others. Towards the end of the epidemic, those persons who had not quitted the country, did not take the fever; it was only the new comers that were affected.

In my practice I had more than twenty cases of adult natives suffering with a fever which exhibited the same character as that which attacked Europeans. The greater part of these persons had made, a greater or less length of time before, (from two to ten years) a voyage to Europe.

I saw no inhabitant of the elevated parts of the island who took the disease upon coming to the city. This was also remarked by other physicians of the preceding epidemics.

If I were governed solely by the results of my own observation, I might affirm that the disease neither prevailed among mulattoes or negroes, (with the exception, however, of some mulatto children:) but I must make it known that I visited principally the white portion of the inhabitants, that the free negroes prescribe for themselves, and that only one or two visits are required for the slaves. The general opinion, however, is, that the negroes and mulattoes were entirely exempt, and if the bills of mortality be consulted, it will be found the mortality among these two classes was not greater than in other months, or in the same months of the preceding year.

*List of Patients furnished to the Hospital by the ships, arranged according to the places from which they came.*

| | | | |
|---|---|---|---|
| Bordeaux, | 35 patients furnished by | 10 ships. | |
| Marseilles, | 30 " " | " 11 " | |
| Havre, | 137 " " | " 34 " | |

I have classed, with those from Havre, three ships from Dunkirk, and two from Nantes. A great number of sailors were treated in the city, whose number it would be impossible to ascertain.

It is shown by this table that the ships from the north furnished a greater number of patients than those from the south. This had also been remarked by MM. Rouchoux and Dariste in the preceding epidemics.

*Constitution.*—Individuals of a sanguine temperament, very muscular, and of a fine complexion, were the most readily and most severely affected; this is indisputable. Nervous persons, of great sensibility, were also unfavourably circumstanced. In running over the list of my patients, and subjecting them to a moral examination (which is quite an easy thing for a physician in a small town) I perceive that a restless, envious mind, and an eagerness to make a fortune, were also unfavourable conditions.

